# A High-Resolution Leaky Coaxial Cable Sensor Using a Wideband Chaotic Signal

**DOI:** 10.3390/s18124154

**Published:** 2018-11-27

**Authors:** Hang Xu, Jun Qiao, Jianguo Zhang, Hong Han, Jingxia Li, Li Liu, Bingjie Wang

**Affiliations:** 1 Key Laboratory of Advanced Transducers & Intelligent Control System, Ministry of Education and Shanxi Province, Taiyuan University of Technology, Taiyuan 030024, China; qiaojun0907@link.tyut.edu.cn (J.Q.); zhangjianguo@tyut.edu.cn (J.Z.); hanhong@tyut.edu.cn (H.H.); lijingxia@tyut.edu.cn (J.L.); liuli01@tyut.edu.cn (L.L.); wangbingjie@tyut.edu.cn (B.W.); 2 College of Physics & Optoelectronics, Taiyuan University of Technology, Taiyuan 030024, China

**Keywords:** perimeter intrusion detection, Boolean-chaos signal, leaky coaxial cable (LCX) sensor

## Abstract

A high-resolution leaky coaxial cable (LCX) sensor for perimeter intrusion detection is proposed and experimentally demonstrated. In our proposed sensor system, a wideband Boolean-chaos signal is used as the probe signal, and a pair of leaky coaxial cables (LCXs) is applied for transmitting the probe signal and receiving the echo signal, respectively. By correlating the chaotic echo signal with its delayed duplicate and comparing the correlation traces before and after intrusion, the intruder can be accurately located. Experimental results demonstrate the proposed sensor can simultaneously detect multiple intruders. The range resolution reaches 30 cm, whilst the dynamic range can achieve 50 dB. In addition, this sensor possesses the excellent anti-interference performance to the noise and uncorrelated chaotic signal, which makes it show robust performance in the detection environment with noise or multiple chaotic LCX sensors cooperation.

## 1. Introduction

Perimeter intrusion detection technologies have been widely applied for high-level security in important places such as railway lines, airport runways, military bases, etc. Commonly used detection techniques include video motion detectors (VMDs) [1,2], infrared sensors [3,4], ground surveillance radars [5,6], and optical fiber sensors [7,8,9], as well as a novel type of leaky coaxial cable (LCX) sensor [10,11]. Compared with the other detection technologies, the LCX sensor has the following significant advantages: high concealability, unlimited installation footprint and independence from environmental impacts (e.g., light, temperature and weather). 

LCX sensors were first introduced for outdoor intrusion detection in the late 1970s. They use two LCXs as transmitting and receiving antennas, which are placed parallel to each other along the perimeter of the monitoring area and shallowly buried underground. A portion of the outer shield is removed from the LCX during its manufacturing process. The openings in the outer conductor facilitate the radiation of an electromagnetic field. If an intruder walks through this invisible electromagnetic field, the field will be disturbed and thus the disturbance signal will trigger an alarm. Until now, LCX sensors have utilized several types of microwave signals as probe signals, including single-tone continuous wave (CW) [12,13], frequency modulation continuous wave (FMCW) [10], stepped frequency continuous wave (SFCW) with phase code modulation [11], radio frequency (RF) pulse [14,15,16,17,18,19,20], and coded pulse sequence [21,22,23,24].

In the early research, LCX sensors formed an electromagnetic monitoring area by transmitting a single-tone continuous wave. It can show whether an intrusion has occurred by detecting the variation of the return waves before and after intrusion. On this basis, Wang et al. proposed a single machine multi-domain perimeter intruder detection system, which transmits continuous waves with different frequencies for different monitoring areas to expand the total monitoring area [13]. Although this type of LCX sensor has a simple hardware structure and measurement principle, it is unable to locate the intruder [10]. 

LCX sensors can also radiate a FMCW [10] or a SFCW with phase code modulation [11] into the surveillance space. Using fast Fourier transform (FFT) techniques, the frequency response is translated into the distance of the intruder along the LCXs. This type of LCX sensor can detect the intruder with one-meter location accuracy [10]. However, its signal generator requires a high-quality direct digital synthesizer (DDS) to achieve low phase noise, fast settling time, and precise frequency control, usually leading to a complex system structure.

Pulsed LCX sensors inject a RF pulse such as 1/2 sine pulse [19] or linear frequency modulation (LFM) pulse [20] into the transmitting LCX to create the electromagnetic surveillance area. An intruder perturbs this electromagnetic field and then causes a reflected signal which is coupled into the receiving LCX. Using quadrature detection technology [17] or comparing the echo signals before and after intrusion [14], this sensor can extract the reflected pulse from the strong direct waves between two LCXs. The delay time between the transmitted pulse and reflected pulse is used to locate the intruder. The pulsed LCX sensor can locate the intruder within one meter [19]. However, limited by the low transmitting pulse-energy levels, this sensor presents a low signal-to-noise ratio (SNR).

In order to enhance the SNR, LCX sensors can transmit a long coded pulse sequence such as pseudo noise (PN) code [21] or complementary orthogonal code based on Golay code [22] instead of a single pulse [24]. Using quadrature detection technology and correlation calculation, the intruder’s distance can be obtained. Moreover, it uses a single processor to monitor two pairs of LCXs (LCXs A and LCXs B) which are connected to each side of the processor. By transmitting the Golay code on LCXs A and complementary Golay code on LCXs B, the monitoring range of this sensor can be extended from one side 400 m to two sides 800 m [23]. However, its location accuracy and location resolution are not enhanced in practice, which are 1 m and 24 m, respectively [24]. 

In realizing low intercept probability and improving range resolution in application of radar [25,26], lidar [27,28] and time domain reflectometry [29,30], chaotic signals has shown significant advantages due to their random characteristics and wideband power spectrum [31,32]. Moreover, chaotic signals have good autocorrelation properties, which makes them have excellent anti-interference performance [33,34]. In 2009, Zhang et al. demonstrated experimentally that wideband Boolean-chaos signals can be generated by autonomous logic gate circuits [35]. Different from the amplitude chaotic signals such as Colpitts chaos and Lorenz chaos, the Boolean-chaos signal is composed of pulses with similar amplitude, and the time interval between the rising edges of adjacent pulses presents a chaotic state. Therefore, as the probe signal of the LCX sensor, the Boolean-chaos signal is more conducive to reduce the requirement of sensor hardware for linear dynamic range. In this paper, we propose a high-resolution intrusion detection sensor based on a wideband chaotic signal and LCXs. The wideband Boolean-chaos signal as the probe signal is transmitted and received by the LCXs, thus forming an electromagnetic field for monitoring intruders. The intruders can be located by correlating the chaotic echo signal with its delayed duplicate and comparing the correlation traces before and after intrusion. Our proposed LCX sensor has the following advantages: (1) The range resolution can reach tens of centimetres by transmitting and receiving the wideband Boolean-chaos signal with LCXs, which is superior to the existing LCX sensors’ meter-scale range resolution. In addition, the wideband Boolean-chaos signal is easily achieved without any complex or costly devices. (2) SNR can be effectively improved by increasing the chaotic correlation length instead of amplifying the signal amplitude as reported in [36]. (3) Anti-interference detection can be realized based on autocorrelation properties of the chaotic signal. The remainder of the paper is organized as follows: In Section 2, the experimental setup is introduced. Section 3 describes the generation and characteristics of Boolean-chaos signal. In Section 4, we show the measure principle. Section 5 estimates the performances of our sensor by analyzing the experimental results. Finally, some discussions and conclusions are outlined in Section 6 and Section 7, respectively.

## 2. Experimental Setup

**Figure 1:**
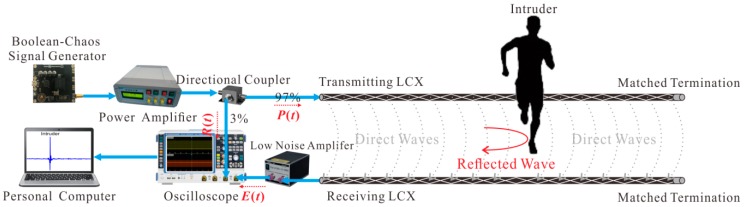
Experimental setup of the LCX sensor utilizing a wideband chaotic signal.

The experimental setup of the LCX sensor utilizing a wideband chaotic signal is shown in Figure 1. The wideband Boolean-chaos signal is generated by a Boolean-chaos signal generator and then amplified by a power amplifier (KG-RF-10, CONQUER, Beijing, China). The amplified chaotic signal is divided into two parts through a 97:3 directional coupler (OH-T-00110-15, A-INFO, Chengdu, China). One part (3% power) serves as a reference signal *R*(*t*) recorded by an oscilloscope (RTO 1024, ROHDE & SCHWARZ, Munich, Germany), and the other part (97% power) as a probe signal *P*(*t*) is radiated by the transmitting LCX (MSLYFYVZ-50-9, Hengteer, Tianjin, China). The echo signal *E*(*t*) is received by the receiving LCX (MSLYFYVZ-50-9, Hengteer, Tianjin, China). The transmitting and receiving LCXs are placed parallel at a certain interval along the perimeter of surveillance area. Matched terminations are provided at the ends of LCXs to terminate the probe and echo signals with the minimal reflection. An electromagnetic field is formed between the transmitting and receiving LCXs as a surveillance area. The echo signal is amplified by a low noise amplifier (SONOMA INSTRUMENT 310, Sonoma Instrument Co., Santa Rosa, CA, USA) and then recorded by the oscilloscope together with the reference signal *R*(*t*). Finally, a personal computer is used for processing data and displaying result. The main parameters of the devices used in our proposed LCX sensor are shown in Table 1.

## 3. Generation and Characteristics of Boolean-Chaos Signal

**Figure 2:**
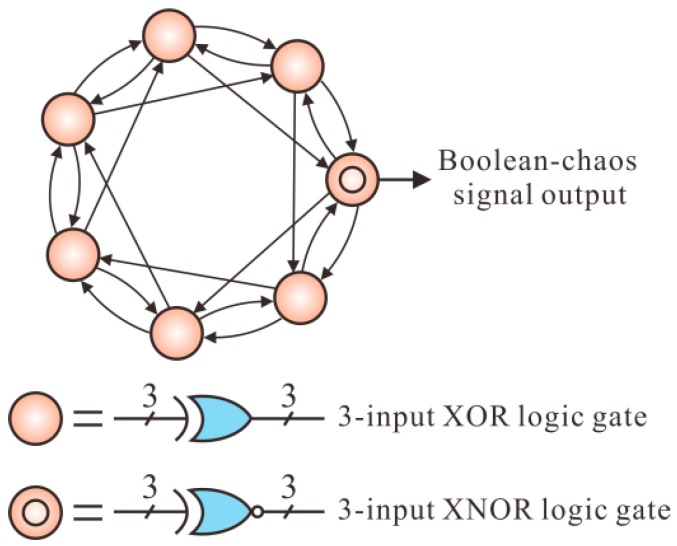
Schematic diagram of the Boolean-chaos signal generator.

An autonomous Boolean network is implemented on a commercial field programmable gate array (FPGA, Cyclone IV EP4CE10F17C8N, Altera, San Jose, CA, USA) as the Boolean-chaos signal generator. Based on non-ideal behavious of logical gates, the Boolean network with a bidirectional ring topology structure can generate the wideband Boolean-chaos signal [37]. As shown in Figure 2, seven nodes are assembled in a bidirectional ring with interval feedback and nearest-neighbour coupling, where six nodes are exclusive-OR (XOR) logical gates with three inputs and three outputs and one node is a XNOR (inverse of the XOR) logical gate with a similar structure. The true tables of XOR and XNOR are shown in [38]. The Boolean-chaos signal is finally output from the XNOR logical gate.

**Figure 3:**
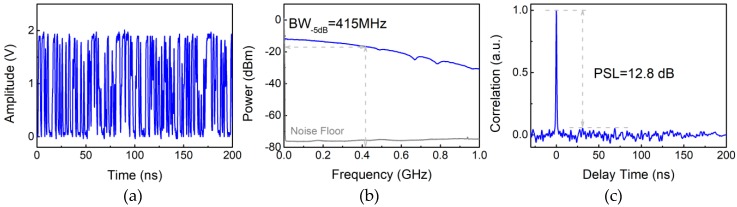
(**a**) Temporal waveform, (**b**) power spectrum, and (**c**) autocorrelation trace of the Boolean-chaos signal.

The characteristics of Boolean-chaos signal used in our sensor are shown in Figure 3. The temporal waveform in Figure 3a indicates the Boolean-chaos signal has a random time interval between the rising edges of adjacent pulses. This chaotic signal exhibits a wide power spectrum with a 5-dB bandwidth (BW) of 415 MHz as shown in Figure 3b. In addition, the Boolean-chaos signal has a delta-function-like autocorrelation trace with an obvious and sharp peak, as plotted in Figure 3c. Here, the peak sidelobe level (PSL) of correlation trace is 12.8 dB. The transmitting power of the Boolean-chaos signal is 21.8 dBm, which is measured by an average power sensor (NRP-Z22, ROHDE & SCHWARZ, Munich, Germany).

## 4. Measure Principle

The proposed intrusion detection sensor uses the LCXs as the antennas to transmit and receive the wideband chaotic signal. The LCX used in our sensor is a sparsely braided LCX [39], which is a typical coupling LCX. The outer conductor of this LCX is generally made up of metal wires woven into many diamond-shaped holes, showing a sparse network. Therefore, electromagnetic waves are radiated or absorbed through these diamond-shaped holes, thus forming an approximate cylindrical electromagnetic field between two LCXs. Compared with the radiation LCX commonly used in existing sensors, the coupling LCX used in our sensor has a wider operating bandwidth and is suitable for transmitting and receiving wideband signals. In addition, because the electromagnetic energy of the coupling LCX is mainly distributed in its near field, our sensor requires a short spacing distance between two LCXs to reduce the transmission loss of wideband signals. 

**Figure 4:**
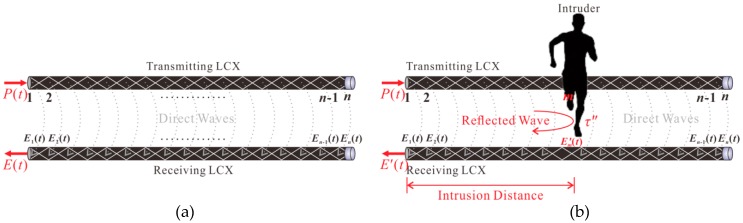
Measurement principle of the proposed LCX sensor (**a**) before and (**b**) after intrusion.

As depicted by Figure 4a, when there is no intruder in the surveillance area, the receiving LCX only receives the direct waves between two LCXs. Assuming that the LCX includes *n* diamond-shaped holes and *i* is any diamond-shaped hole, the echo signal *E*(*t*) without the intruder can be expressed as:

(1) $$E\left(t\right)=\sum_{i=1}^{n}E_{i}\left(t\right)$$

where *E_i_*(*t*) is the direct wave received by the *i*-th hole on the receiving LCX. A calibration trace *C*(*τ*) is obtained by correlating the reference signal *R*(*t*) and echo signal *E*(*t*) without intruder, which is given by:

(2) $$C\left(\tau \right)=E\left(t\right)⊗R\left(t\right)=\sum_{i=1}^{n}E_{i}\left(t\right)⊗R\left(t\right)=\sum_{i=1}^{n}\left[E_{i}\left(t\right)⊗R\left(t\right)\right]$$

where *τ* is the delay time between *E*(*t*) and *R*(*t*), which represents the set of roundtrip times of all direct waves, and ⊗ denotes the correlation operator. It can be seen that *C*(*τ*) is a superposition of correlation traces caused by the direct waves.

If an intruder crosses the LCXs, the part of probe signal will be reflected by the intruder and mainly received by the *m*-th hole on the receiving LCX together with direct waves, as plotted in Figure 4b. The echo signal *E′*(*t*) with an intruder can be written as:

(3) $$E^{\prime }\left(t\right)=\sum_{i=1}^{m-1}E_{i}\left(t\right)+\sum_{i=m+1}^{n}E_{i}\left(t\right)+{E^{\prime }}_{m}\left(t\right)\approx E\left(t\right)+{E^{\prime }}_{m}\left(t\right)$$

where ${E^{\prime }}_{m}\left(t\right)$ is the reflected signal from the intruder and received by the *m*-th hole on the receiving LCX. A intrusion trace $C^{\prime }\left(\tau^{\prime }\right)$ is obtained by correlating the reference signal *R*(*t*) and echo signal *E′*(*t*) with an intruder, as expressed below:

(4) $$C^{\prime }\left(\tau^{\prime }\right)=E^{\prime }\left(t\right)⊗R\left(t\right)=\left[E\left(t\right)+{E^{\prime }}_{m}\left(t\right)\right]⊗R\left(t\right)=C\left(\tau \right)+{E^{\prime }}_{m}\left(t\right)⊗R\left(t\right)$$

where *τ′* is the delay time between *E′*(*t*) and *R*(*t*), which expresses the set of roundtrip times of all direct waves and the reflected wave. *C′*(*τ′*) is a superposition of correlation traces caused by direct waves and the reflected wave.

The correlation trace Δ*C*(*τ″*) caused by the reflected wave is obtained by subtracting the calibration trace *C*(*τ*) from the intrusion trace *C′*(*τ′*), as shown below:

(5) $$\Delta C\left(\tau^{\prime \prime }\right)=C^{\prime }\left(\tau^{\prime }\right)-C\left(\tau \right)={E^{\prime }}_{m}\left(t\right)⊗R\left(t\right)=\delta \left(t-\tau^{\prime \prime }\right)$$

where *τ″* is the roundtrip time between the sensor and intruder along the LCXs. The intruder’s distance is determined by extracting the correlation peak position and calculating *υτ″*/2, where *υ* is the propagation velocity of the electromagnetic wave in the LCXs.

## 5. Experimental Results

### 5.1. Detection of Single Intruder

**Figure 5:**
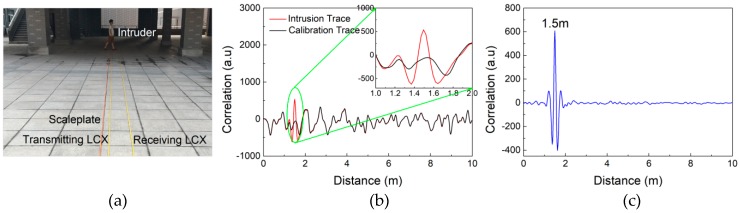
(**a**) Experimental scene of intrusion detection. (**b**) Detection results before and after intrusion. (**c**) Final intrusion detection result.

Figure 5a shows the experimental scene of an intruder passing through the monitoring area consisting of two LCXs. The LCXs are placed parallel on dry ground with 0.4-m spacing distance, and a scaleplate is placed parallel to the LCXs to calibrate the actual distance of the intruder. Figure 5b shows the detection results before and after intrusion, named intrusion trace (red curve) and calibration trace (black curve), respectively. They reveal a significant change at the distance of the intruder. By subtracting the calibration trace from the intrusion trace, a correlation peak at 1.5 m representing the distance of the intruder is obtained as shown in Figure 5c.

**Figure 6:**
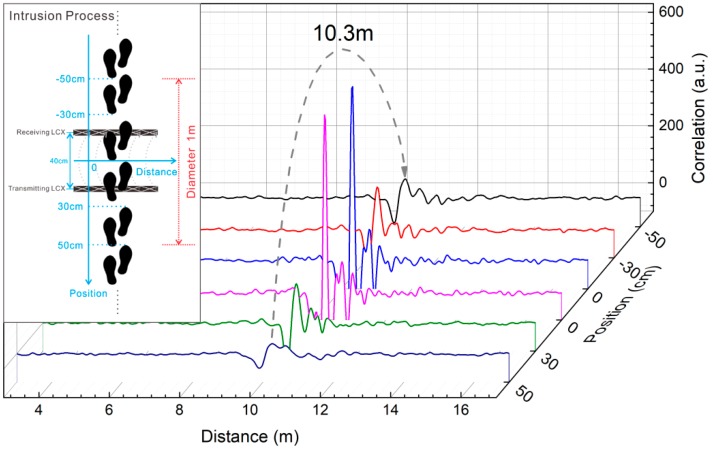
Detection results of intrusion process.

The intrusion process is further monitored using our sensor. The process of the intruder crossing the LCXs is simply shown in the inset of Figure 6. Figure 6 shows that as the intruder approaches firstly, then crosses, and finally leaves the LCXs, the correlation peak value increases firstly and reaches the maximum corresponding to the first two stages of intrusion process, then it decreases demonstrating the leaving process. This changing of peak value is because that when the intruder comes close to the LCXs, a stronger reflected signal caused by the intruder can be obtained with a higher correlation peak. Therefore, by monitoring the highest correlation peak occurs when the intruder crosses the LCXs, we can judge whether there is an intrusion or not. 

**Figure 7:**
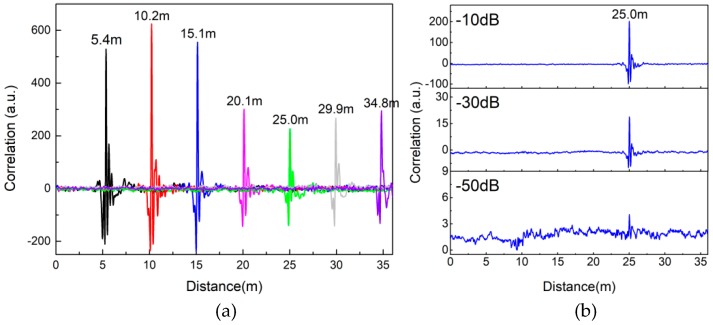
(**a**) Detection results of different intrusion distances. (**b**) Dynamic range analysis of our sensor.

Figure 7a gives the detection results of an intruder crossing the LCXs at different distances. The results show that the intruder can be clearly located by the correlation peak position in the range of 35 m. Besides, with the increase of detection distance, the correlation peak value presents an overall downward trend as a whole. Due to the non-uniform distribution and inconsistent size of the diamond-shaped holes in the LCXs, the correlation peak value has some fluctuation, but this does not affect the detection effect. Limited by the size of the experimental site, the maximum detectable distance obtained experimentally is 35 m. In order to estimate the maximal detectable range, we investigate the available dynamic range of our sensor. Choosing the intrusion detection result at 25.0 m as the research object, we decrease the transmitting power by 10 dB, 30 dB and 50 dB, respectively. The corresponding detection results show the correlation peak value declines with the decrease of the transmitting power, as shown in Figure 7b. However, we notice that even if the transmitting power decreases by 50 dB, the intrusion distance can still be judged by the correlation peak. With the further reduction of transmitting power, the correlation peak is submerged in the background noise and the intruder becomes undetectable. Therefore, it can be concluded that the dynamic range of our sensor can reach 50 dB. The attenuation constant of the LCX used in our experiments is 8.8 dB/100 m for the wideband chaotic signal, which is measured by the average power sensor. Therefore, in the case of one intruder, the maximum detectable distance of our sensor is estimated to be 310 m (50 dB/8.8 dB × 100 m/2 + 25.0 m ≈ 310 m).

**Figure 8:**
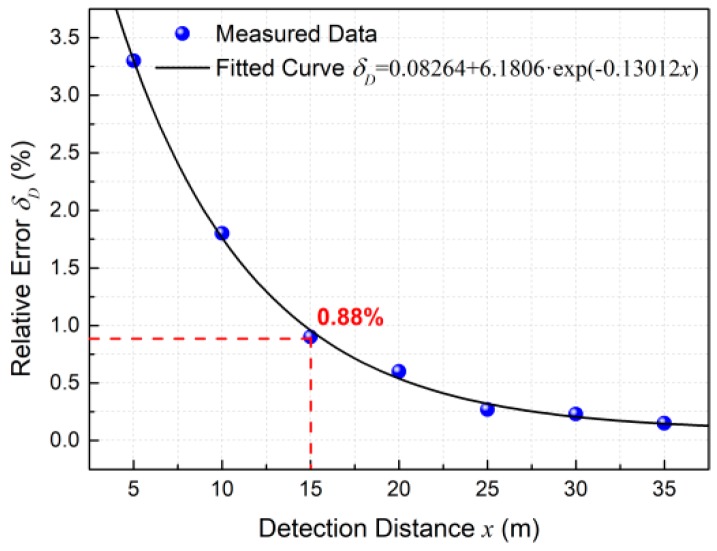
Variation of relative error *δ_D_* with the increase of detection distance *x*.

The relative error *δ_D_* is used to measure the accuracy of intrusion detection in our experiment, as defined below:

(6) $$\delta_{D}=\frac{\left|x-D\right|}{D}\times 100\%$$

where *x* is the detection distance of the intruder measured by our sensor, *D* is the actual distance of the intruder measured by the scaleplate. The measured data and fitted curve in Figure 8 show the relative error *δ_D_* decreases exponentially with the increase of detection distance *x*. The relative error of measurement is 0.88% when the distance reaches 15 m, and it further slowly decreases with the increase of detection distance. 

### 5.2. Detection of Multiple Intruders

**Figure 9:**
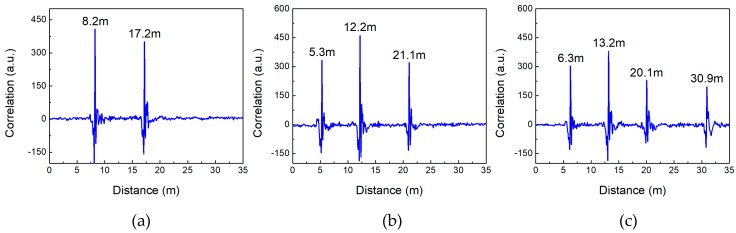
Detection results of (**a**) two intruders, (**b**) three intruders and (**c**) four intruders.

Figure 9 shows the detection results of multiple intruders crossing the LCXs simultaneously. From Figure 9a–c, two intruders, three intruders as well as four intruders are simultaneously located, respectively. The detectable number of intruders can further increase. It indicates that our sensor has the ability to detect multiple intruders simultaneously. 

To measure the range resolution, we choose two intruders as research targets. They crosses the LCXs simultaneously with a series of different spacing distances, and one of them intrudes at a fixed distance of 15.1 m. 

**Figure 10:**
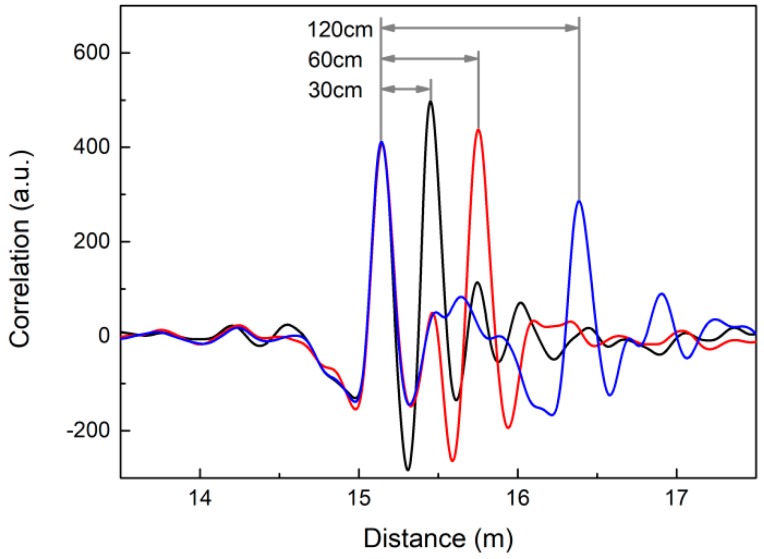
Detection results of two intruders with different spacing distances.

Figure 10 shows the detection results of two intruders with different spacing distances, that are 120 cm, 60 cm and 30 cm respectively. The experimental results demonstrate that two intruders with 30-cm spacing distance can be clearly defined from two correlation peaks. In theory, the range resolution is defined as *c*/2*B*, where $c=c_{0}/\sqrt{\mu_{r}\varepsilon_{r}}=0.83c_{0}$, $c_{0}=3.0\times 10^{8}\;m/s$, and *B* is the signal bandwidth. Therefore, the 415-MHz bandwidth of Boolean-chaos signal corresponds to the range resolution of 30 cm. The 30-cm range resolution we obtain in experiments is accordance with theoretical expectation, which is superior to the existing LCX sensors’ range resolution of meter-scale. For example, the range resolution of the LFM pulsed LCX sensor reported in [20] is 6.64 m.

### 5.3. Anti-Interference Analysis

**Figure 11:**
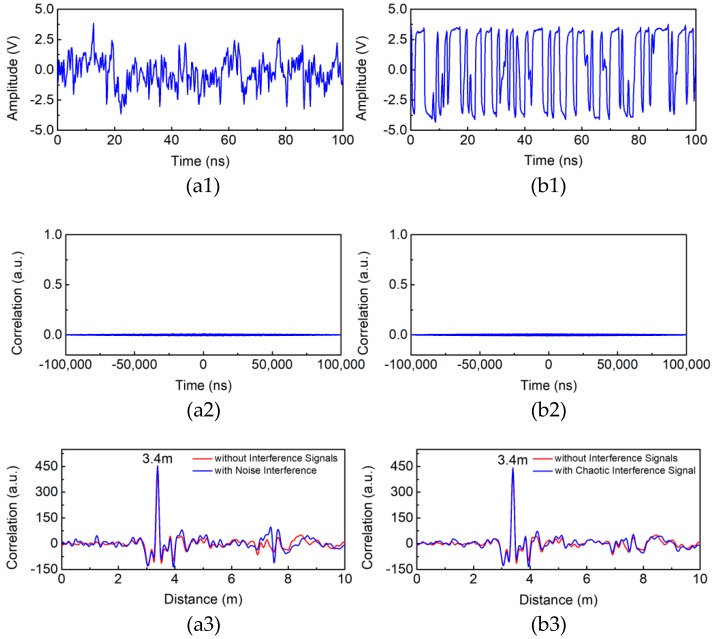
Temporal waveform of (**a1**) noise and (**b1**) chaotic interference signal. Crosscorrelation traces of (**a2**) noise and chaotic reference signal, (**b2**) chaotic interference signal and chaotic reference signal. Comparison of detection results with and without (**a3**) noise and (**b3**) chaotic interference signal.

Compared with aforementioned probe signals of LCX sensors such as single-tone continuous wave, FMCW, and RF pulse, the Boolean-chaos signal shows stronger anti-interference abilities to external electromagnetic interferences benefiting from its merits in autocorrelation properties. The anti-interference performance of our sensor is discussed in this part. After adding one more LCX to transmit interference signals into the monitoring area, we analyze the influence of interference signals on detection results. The added LCX is parallel and adjacent to the transmitting LCX. The noise and uncorrelated Boolean-chaos signal as interference signals are shown in Figure 11(a1,b1) respectively. These signals are used to simulate the detection environment with noise or multiple chaotic LCX sensors working together. The transmitting power of interference signals is 21.8 dBm, which is the same as that of chaotic probe signal. Figure 11(a2,b2) show crosscorrelation traces of interference signals and chaotic reference signal. It is obviously that there is no peak in crosscorrelation traces, which indicates that these interference signals are uncorrelated with the chaotic reference signal. The detection results of adding the interference signals (blue curves) or not (red curves) are shown in Figure 11(a3,b3) respectively. 

As expected, the interference signals do not change the position and value of the correlation peak representing the intruder’s distance. Experimental results show that our chaotic LCX sensor has a good resistance to noise, which makes it be suited for noise environment and has a potential application in expanding the monitoring area by multiple chaotic LCX sensors cooperation.

## 6. Discussions

As mentioned earlier, an approximate cylindrical electromagnetic field is formed between two LCXs. Figure 6 indicates that an intruder can be detected by the correlation peak even though the intruder has a 0.3-m distance from the LCXs, whilst the spacing distance of two LCXs is 0.4 m. So the diameter of the cylindrical electromagnetic field is 1 m. According to aforementioned measurement results of dynamic range, the maximum detectable distance of our sensor, that is the length of the cylindrical electromagnetic field, is estimated to be 310 m. The LCXs are placed parallel on ground, causing half of the cylindrical electromagnetic field to be exposed on ground. Therefore, the sensing volume of our LCX sensor is estimated as 122 m^3^ (3.14 × 0.5^2^ × 310/2 m^3^).

At the present stage, chaotic signal analysis in our experiments only provides the intrusion distance. The correlation peak height reflects the intensity of reflected signal which may relate to the size of the intruder. We have reason to consider that even under the same intrusion distance, a larger intruder will induce a stronger reflected signal and a higher correlation peak. Therefore, small animals as nuisance alarms and human may be distinguished by setting a suitable threshold of the correlation peak height in advance. This will be discussed in detail in our future work.

## 7. Conclusions

In conclusion, we have proposed and experimentally demonstrated a high-resolution chaotic LCX sensor for perimeter intrusion detection. The wideband chaotic signal is firstly applied as the probe signal in the LCX sensor, and the intruder is located by correlating the chaotic echo signal with its delayed duplicate and comparing the correlation traces before and after intrusion. Experimental results demonstrate the proposed sensor can realize the simultaneous detection of multiple intruders. The range resolution and dynamic range can reach 30 cm and 50 dB, respectively. The maximum detectable distance is estimated to be 310 m when the transmitting power is 21.8 dBm. The relative error is less than 1% when the detection distance exceeds about 15 m. Additionally, this sensor possesses the excellent anti-interference performance to the noise as well as uncorrelated chaotic signal, which makes it perform superbly in noise or multiple chaotic LCX sensors cooperation environment.

## Figures and Tables

**Table 1:** The main parameters of the devices used in our proposed LCX sensor.

Devices	Pass-Band/Bandwidth	Other Parameters
Power amplifier	75 Hz–10 GHz	Max gain: 25 dB
Directional coupler Low noise amplifier Oscilloscope LCXs	1 MHz–1 GHz 9 kHz–1 GHz 2 GHz ≤450 MHz	Coupling degree: 15 dB Max gain: 32 dB Sampling rate: 10 GSa/s
